# Shared prognostic information in amyotrophic lateral sclerosis – systematic assessment of the patients’ perception of neurofilament light chain and the ALS functional rating scale

**DOI:** 10.1186/s42466-024-00363-y

**Published:** 2025-02-06

**Authors:** Lukas Maximilian Möhwald, André Maier, Torsten Grehl, Ute Weyen, Patrick Weydt, René Günther, Paul Lingor, Bettina Göricke, Susanne Petri, Julian Grosskreutz, Matthias Boentert, Isabell Cordts, Jochen H. Weishaupt, Johannes Dorst, Christoph Münch, Thomas Meyer, Petra Baum

**Affiliations:** 1 Medical Faculty, Department of Neurology, Universitätsklinikum Leipzig, Liebigstraße 20, 04103 Leipzig, Germany; 2 Department of Neurology, Center for ALS and other Motor Neuron Disorders, Charité- Universitätsmedizin Berlin, Augustenburger Platz 1, 13353 Berlin, Germany; 3 Department of Neurology, Center for ALS and other Motor Neuron Disorders, Alfried Krupp Krankenhaus, Essen, Germany; 4 Department of Neurology, Center for ALS and other Motor Neuron Disorders, Berufsgenossenschaftliches Universitätsklinikum Bergmannsheil, Bochum, Germany; 5 Department for Neuromuscular Disorders, Bonn University, Bonn, Germany; 6 Research Site Bonn, Deutsches Zentrum für Neurodegenerative Erkrankungen (DZNE), Bonn, Germany; 7 Department of Neurology, Technische Universität Dresden, Dresden, Germany; 8 Deutsches Zentrum für Neurodegenerative Erkrankungen, Research Site Dresden (DZNE), Dresden, Germany; 9 Department of Neurology, School of Medicine, Klinikum rechts der Isar, Technical University of Munich, Munich, Germany; 10 Department of Neurology, Universitätsmedizin Göttingen, Göttingen, Germany; 11 Department of Neurology, Hannover Medical School, Hannover, Germany; 12 Precision Neurology in Neuromuscular and Motoneuron Diseases, Cluster of Excellence Precision Medicine in Inflammation (PMI), University of Lübeck, Lübeck, Germany; 13 Department of Neurology, Universitätsklinikum Münster, Münster, Germany; 14 Department of Neurology, Division for Neurodegenerative Diseases, Mannheim Center for Translational Medicine, University Medicine Mannheim, Heidelberg University, Mannheim, Germany; 15 Department of Neurology, Ulm University, Ulm, Germany; 16 Ambulanzpartner Soziotechnologie APST GmbH, Berlin, Germany

**Keywords:** Amyotrophic lateral sclerosis, Amyotrophic lateral sclerosis functional rating scale - revised, Neurofilament light chain, Fear of progression, Perception

## Abstract

**Background:**

In amyotrophic lateral sclerosis (ALS), neurofilament light chain (NfL) was introduced as a prognostic biomarker. More recently, NfL values can be shared on the patient’s ALS app. Also, the ALS functional rating scale (ALSFRS-R) is an established patient-reported assessment of disease progression. The scale can be obtained during clinic visits or remotely. However, few systematic data are available on the patients’ perception of prognostic information about NfL and ALSFRS-R and the remote sharing of these data.

**Methods:**

In a multicenter study, 149 ALS patients were assessed for their perception of shared information about NfL and ALSFRS-R using an investigator-designed survey and established questionnaires. The recommendation of NfL and ALSFRS-R to fellow patients was assessed using the Net Promoter Score (NPS). Burden by shared information was investigated in two distinct settings: (1) clinic information when receiving results on NfL and/or ALSFRS-R during clinic visits and (2) remote information about NfL values and self-rating of the ALSFRS-R via the ALS app. General anxiety was measured by the Fear of Progression Questionnaire – Short Form (FoP-Q-SF).

**Results:**

Information about NfL and ALSFRS-R, respectively (*n* = 149), were regarded as relevant for patients themselves (75.2% and 77.2%) and for research (98% and 96%). The NPS showed a high recommendation rate for NfL (+ 21) and ALSFRS-R (+ 26). Only a minority of patients perceived shared information about NfL as burdensome, with a lower burden in the clinic setting (*n* = 1, 4.2%) than in the remote setting (*n* = 8, 12%; *p* = 0.015). Remote digital assessment of the ALSFRS-R was well received, with a reported burden in 9.8% (*n* = 9) of the participants. The FoP-Q-SF revealed fear of progression in 40% of the respondents (*n* = 60).

**Conclusions:**

This study underscored the relevance of information about NfL and ALSFRS-R from the patient’s perspective. Furthermore, patients proved to appreciate the relevance of this data for ALS research. Sharing information about NfL or ALSFRS-R was rarely perceived as burdensome even in a remote setting using the ALS app. These findings pave the way for further development of the patient-centered approach to sharing prognostic information in ALS.

## Background

Amyotrophic lateral sclerosis (ALS) is a severe neurodegenerative disease that is associated with an increasing loss of motor functions [1]. Various methods have been established to assess the variable course and the individual prognosis of people with ALS, such as the measurement of neurofilament light chain (NfL) and the ALS functional rating scale in its revised form (ALSFRS-R) [2]. NfL is a biomarker of ALS that is highly correlated with disease progression as measured by the ALS progression rate [3–5]. More specifically, increased serum and CSF NfL values were shown to be associated with aggressive disease progression and shorter life expectancy [6, 7]. The ALSFRS-R is an established questionnaire for the assessment of disease severity and progression [8]. The scale can be obtained either by physicians or patients alike – during clinic visits or remotely via telephone [9] and remote digital assessment [10]. Its consistent administration across all assessment scenarios using an annotated version conforms to a national consensus in Germany [11]. The ALSFRS-R evaluates 12 different functional areas typically affected by ALS [12]. The monthly change in the ALSFRS-R score is a patient-reported outcome measure of ALS progression and is considered a prognostic factor for the further course of disease and survival [13]. Both NfL and ALSFRS-R are of interest for health care professionals and for patients to gain insights in the individual prognosis that is often in the range of 2–4 years but shows great variability [4]. Digital applications such as the mobile smartphone application “ALS app” facilitate the remote digital self-assessment of the ALSFRS-R [14]. Only recently, the NfL diagram was included in the ALS app that displays NfL values being generated in a multicenter biomarker program. The remote sharing of NfL and ALSFRS-R information on mobile devices creates a novel constellation in which patients are exposed to prognostic information – apart from a conventional clinic consultation [14]. Given the novelty of digital data sharing concepts, few data are available on the patients’ perception of remotely shared prognostic information on NfL and ALSFRS-R. Positive aspects of shared information, such as improving knowledge about the disease, must be weighed against potential burdens, such as uncertainty and anxiety [15, 16].

When analyzing the patients’ perspective on NfL and ALSFRS-R, this study aims (1) to assess patients’ perceptions of shared information about NfL and ALSFRS-R; (2) to determine the recommendation of NfL and ALSFRS-R to fellow ALS patients using the Net Promoter Score (NPS); (3) to investigate and compare the burden of sharing information of NfL and ALSFRS-R in a remote and clinic setting; and (4) to explore the general anxiety level as measured by based the fear of progression (FoP) questionnaire.

## Methods

### Study design

The patient survey was conducted as part of a multicenter prospective observational study, which was carried out according to the STROBE criteria [17, 18].

### Participants

The following inclusion criteria were applied: (1) diagnosis of ALS according to the Gold Coast criteria [19]; (2) consent to participate in the study and to complete the data collection form, and (3) consent to electronic data collection via a digital research platform (group of remote digital assessment).

### Measurement of NfL

To determine the individual NfL value, a blood sample was taken during the patient’s visit to a specialized ALS center in Germany. A serum collection tube was used, which could be clearly assigned to the patient. NfL was measured in the ALS outpatient clinic of Charité - Universitätsmedizin Berlin using highly sensitive analysis methods [20], whereby a certain number of samples were collected and then analyzed together.

### Setting

#### Information sharing about NfL and ALSFRS-R

**Fig. 1:**
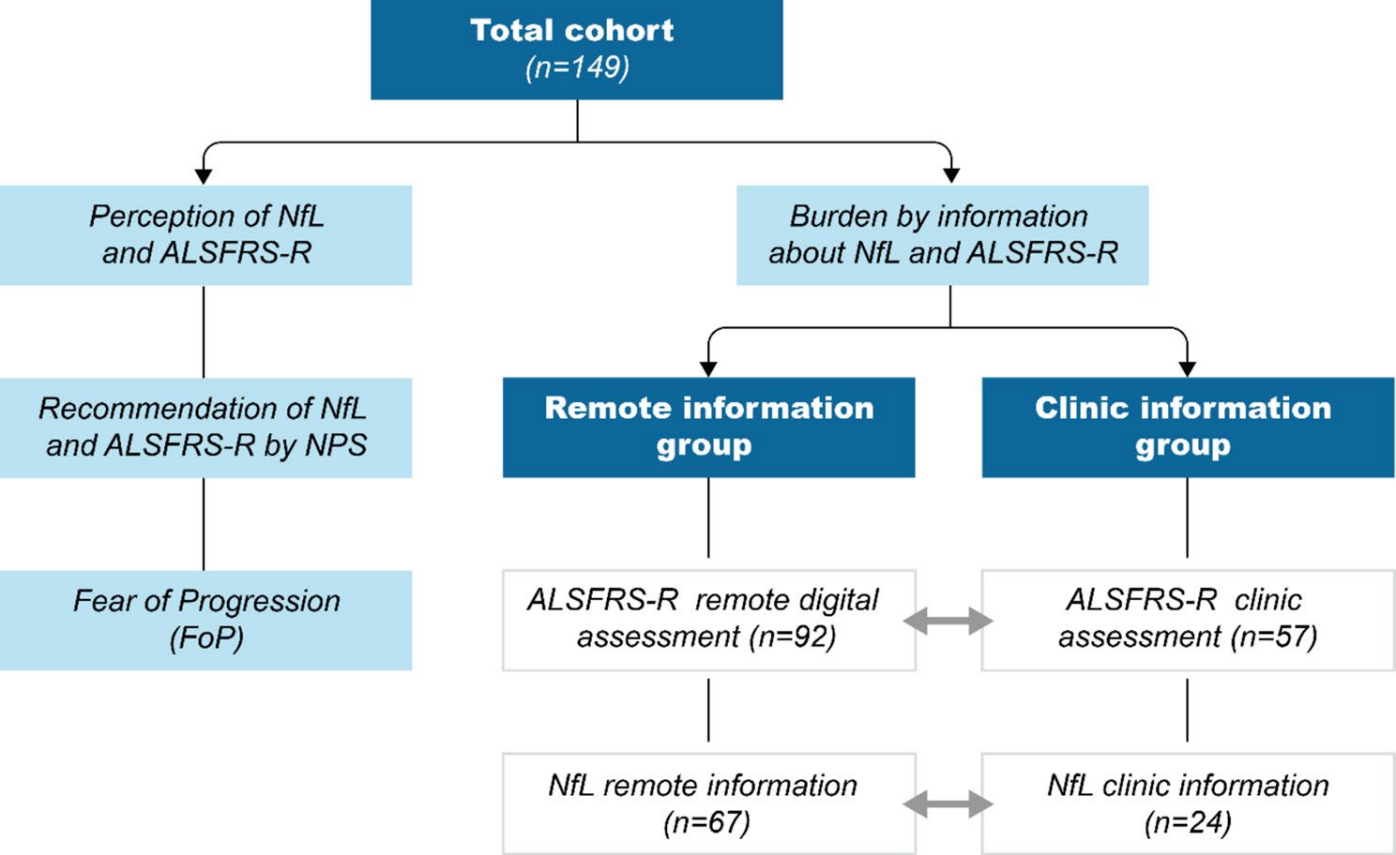
Overview of the study design. Patients provided information on their perception of NfL and ALSFRS-R, including the respective recommendation rate (NPS) and a possible fear of progression (FoP). To evaluate the psychological burden by information about NfL and ALSFRS-R, the overall cohort was divided into (1) a “remote information group” and (2) a “clinic information group”. *n* Number of patients; *NfL* Neurofilament Light Chain; *ALSFRS-R* ALS Functional Rating Scale Revised; *NPS* Net Promotor Score; *FoP-Q-SF* Fear of Progression Questionnaire – Short Form

Information about NfL and ALSFRS-R was shared in two distinct settings: (1) remote information about NfL values and self-rating of the ALSFRS-R and (2) clinic information when receiving results on NfL and/or ALSFRS-R during clinic visits. The clinic setting represented the standard of care in which information about NfL and ALSFRS-R was shared during regular visits. The remote setting was offered in addition to the standard of care, as patients were invited to perform a monthly remote digital assessment of the ALSFRS-R on a mobile application (“ALS app”). The ALS app included the NfL diagram that shows the results of the patient´s NfL measurements in relation to the age-adjusted reference values. The ALS app displayed NfL values in the short term after the completion of NfL measurements, as it was synchronized with the NfL study software. Thus, remote sharing of NfL information was commonly faster than the (additional) notification of NfL results during clinic visits (Fig. 1).

#### Data collection

Data were collected between October 2022 and October 2023. Two approaches were used for data collection: (1) a printed questionnaire for the direct patient survey (print survey) and (2) an online version of the questionnaire (online survey), which was distributed via online survey software that was part of the research platform “APST” [21]. Demographic and clinical data were obtained from the electronic health records of the participating ALS centers.

### Protocol approvals and registrations

The study protocol was approved by the Medical Ethics Committee of Charité - Universitätsmedizin Berlin, Germany, under the number EA2/168/20. Informed consent was obtained from all individual participants included in the study.

### Variables

#### Perception of NfL

Perception of the NfL biomarker was assessed with four questions and five answer options, respectively.

Questions:

Q 1: “Do you perceive the NfL as relevant for yourself?”

Q 2: “Do you perceive the NfL as relevant for research?”

Q 3: “Do you have interest in the results of your NfL?”

Q 4: “Do you have information gain from the NfL?”

Answer options: (1) very high agreement, (2) high agreement, (3) some agreement, (4) low agreement, (5) no agreement.

#### Burden by shared information about NfL

Patients with a known NfL value were asked about the burden by shared information on NfL in two distinct settings: (1) a “remote information group” that gained access to their individual NfL values being displayed on a mobile application named “ALS app”; and (2) a “clinic information group” that received their results on NfL during clinic visits. Burden by the shared information about NfL was assessed with one question (Q5): “Do you perceive burden caused by the information about NfL?”.

#### Perception of the ALSFRS-R

The patient’s perception of the ALSFRS-R was assessed with four questions and five answer options, respectively.

Questions:

Q 1: “Do you perceive the ALSFRS-R as relevant for yourself?”

Q 2: “Do you perceive the ALSFRS-R as relevant for research?”

Q 3: “Do you have interest in the results of your ALSFRS-R?”

Q 4: ”Do you have information gain from the ALSFRS-R?”

Answer options: (1) very high agreement, (2) high agreement, (3) some agreement, (4) low agreement, (5) no agreement.

#### Burden by shared information about ALSFRS-R

A question about burden caused by ALSFRS-R remote digital assessment was asked of all patients who had relevant previous experience (Q5): “Do you perceive burden caused by the self-assessment of the ALSFRS-R?”.

Answer options: (1) very high agreement, (2) high agreement, (3) some agreement, (4) low agreement, (5) no agreement.

#### Recommendation of NfL and ALSFRS-R

The Net Promotor Score (NPS) was used to determine the degree of recommendation of the NfL biomarker and the ALSFRS-R to fellow ALS patients [22]. This metric was calculated based on responses to a single question: “How likely is it that you would recommend NfL (ALSFRS-R) to a fellow ALS patient?” Possible answers ranged from 0 (very unlikely recommendation) to 10 (very likely recommendation) points. Patients were considered as “promoters” (10 or 9 score points), “indifferent” (8 or 7 points), or “detractors” (6 to 0 points). The NPS is calculated by subtracting the percentage of detractors from the percentage of promoters. NPS can have values between + 100 and − 100, whereby an NPS greater than zero is regarded as a supporting recommendation.

#### Fear of progression (FoP)

The “Fear of Progression Questionnaire - Short Form (FoP-Q-SF)” is based on 12 questions addressing five domains of potential worries about the future (affective reactions, relationship/family problems, occupation and loss of autonomy) [23]. Study participants were asked to indicate the frequency of their respective worries about the future on a five-point Likert scale from 1 (never) to 5 (very often) [24]. A total score between 12 and 60 points was calculated by adding up the individual points. Fear of progression (FoP) was defined from a cut-off value of ≥ 34 points [25, 26].

### Statistical analysis

The statistical analysis was performed using SPSS (version 29.0). The descriptive statistics included frequency (%), mean, median and standard deviation (±). Differences in mean values between groups were assessed by t-test for metric scale levels and normal distribution; otherwise, the Mann-Whitney U test was used. The p-values were given with a confidence interval of 95%.

## Results

### Characteristics of the total cohort

A total of 149 patients were included in the study. The demographic and clinical characteristics of the study participants can be found in Table 1. 43% were female (*n* = 64) and 57% were male (*n* = 85). The mean age of the patients was 62 years, whereas the mean time since the onset of symptoms was 58.7 months. In 57% of the patients (*n* = 85) the disease started in the limbs and in 18% (*n* = 27) in the bulbar region, in a quarter of the cases (*n* = 37) no data were available. The mean ALSFRS-R total score was 31.6 points (maximum of 48 points).

**Table 1:** Characteristics of the study cohort

Characteristics	Parameters
Gender (F/M)	43% (n=64) / 57% (n=85)
Age (years) (mean±SD)	62.04 (±10.5)
Type of onset (spinal/bulbar/no data)	57% (n=85) / 18% (n=27) / 25% (n=37)
Disease duration (months) (mean±SD)	58.73 (±57.1)
ALSFRS-R (mean ± SD)	31.64 (±10.5)
NfL, remote information (yes/no)	73.6% (n=67) / 26.4% (n=24)
ALSFRS-R, remote information (yes/no)	61.7% (n=92) / 38.3% (n=57)

*Abbreviations* n Number of patients; SD standard deviation; ALSFRS-R ALS Functional Rating Scale Revised; NfL Neurofilament Light Chain

### Characteristics of clinic and remote information groups

The “clinic information group” included 57 patients who received information about the ALSFRS-R score, of which 24 patients were notified about the NfL value. The “remote information group” consisted of 92 patients who had completed remote digital assessment of the ALSFRS-R. Of these, 67 patients also received information about NfL via the ALS app. The NfL-clinic information group (*n* = 24) was similar to the NfL-remote information group (*n* = 67) in terms of disease duration (*p* = 0.535), age (*p* = 0.252) and ALSFRS-R score (*p* = 0.662).

### Perception of NfL and ALSFRS-R

**Fig. 2:**
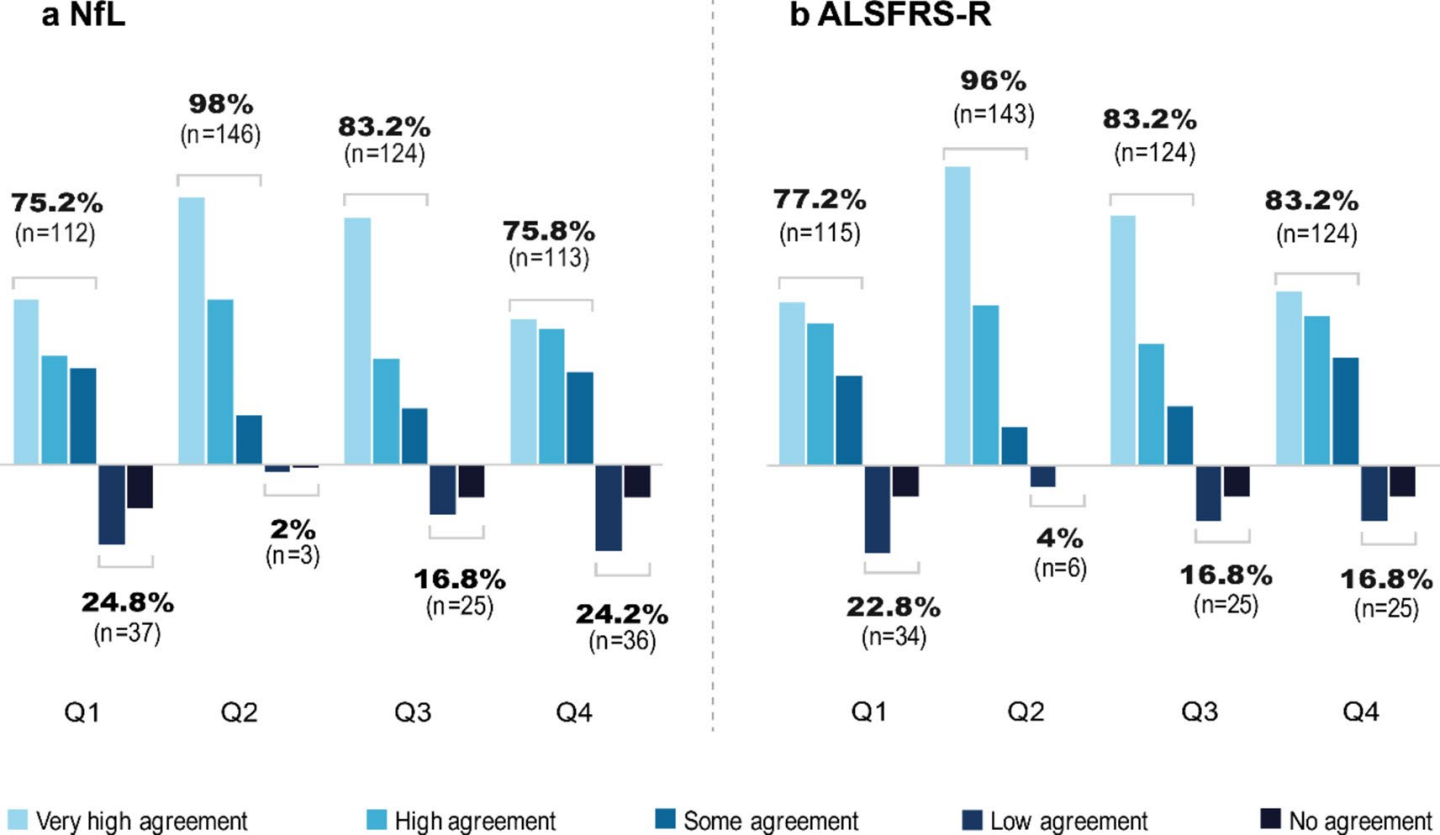
Perception of information about NfL and ALSFRS-R. **a NfL.** Four questions addressed the perception of shared information about NfL: **Q1** “Do you perceive the NfL as relevant for yourself?”; **Q2** “Do you perceive the NfL as relevant for research?”; **Q3** “Are you interested in the results of your NfL?”; and **Q4** “Have you gained any information from the NfL?”. **b** ALSFRS-R. Four questions addressed the perception of shared information about ALSFRS-R: **Q1** “Do you perceive the ALSFRS-R as relevant for yourself?”; **Q2** “Do you perceive the ALSFRS-R relevant for research?”; **Q3** “Do you have interest in the results of your ALSFRS-R?”; and **Q4** “Do you have information gain from the ALSFRS-R?”. *n* Number of patients, *NfL* Neurofilament Light Chain; *ALSFRS-R* ALS Functional Rating Scale Revised

Of the total cohort (*n* = 149), 75.2% (*n* = 112) perceived NfL to be relevant for the patients themselves. Almost all patients (98%; *n* = 146) recognized its relevance for research. A total of 83.2% (*n* = 124) were very interested in NfL, with 75.8% of patients (*n* = 113) perceiving an information gain. Concerning the ALSFRS-R, 77.2% of patients (*n* = 115) perceived the assessment of the scale as relevant from the patient´s perspective. Most patients were convinced of the relevance for research (96%; *n* = 143). A total of 83.2% (*n* = 124) of the study participants had high interest in the scale. 83.2% (*n* = 124) perceived an information gain about the course of the disease. Results are shown in Fig. 2.

### Recommendation of NfL and ALSFRS-R by NPS

**Fig. 3:**
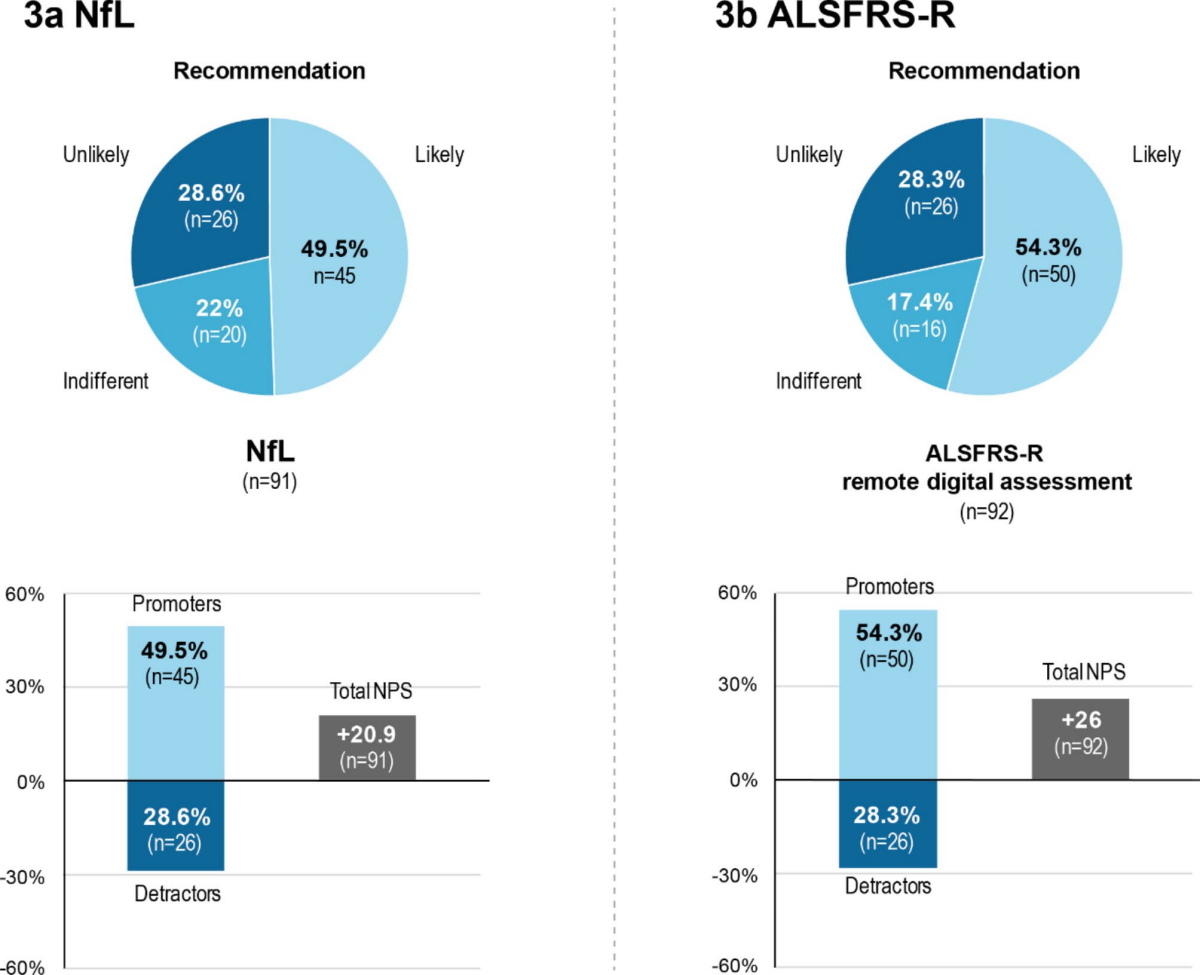
Recommendation of NfL and ALSFRS-R. The degree of recommendation was determined using the Net Promotor Score (NPS). This metric was calculated based on responses to a single question: “How likely is it that you would recommend NfL (ALSFRS-R) to a fellow ALS patient?”. Values between + 100 and − 100 were possible. *n* Number of patients; *NfL* Neurofilament Light Chain; *ALSFRS-R* ALS Functional Rating Scale Revised

Patients with shared information about NfL (*n* = 91) included promoters in 49.5% (*n* = 45), contrasted by 28.6% (*n* = 26) of detractors whereas 22% (*n* = 20) were indifferent. The total NPS (subtraction of detractors from promotors) was + 20.9, indicating a positive recommendation rate. Of the patients performing remote digital assessment of the ALSFRS-R (*n* = 92), 54.3% (*n* = 50) were promoters, followed by 28.3% (*n* = 26) who were detractors and 17.4% (*n* = 16) who were indifferent. The total NPS was + 26 (Fig. 3).

### Burden by information about NfL and ALSFRS-R

**Fig. 4:**
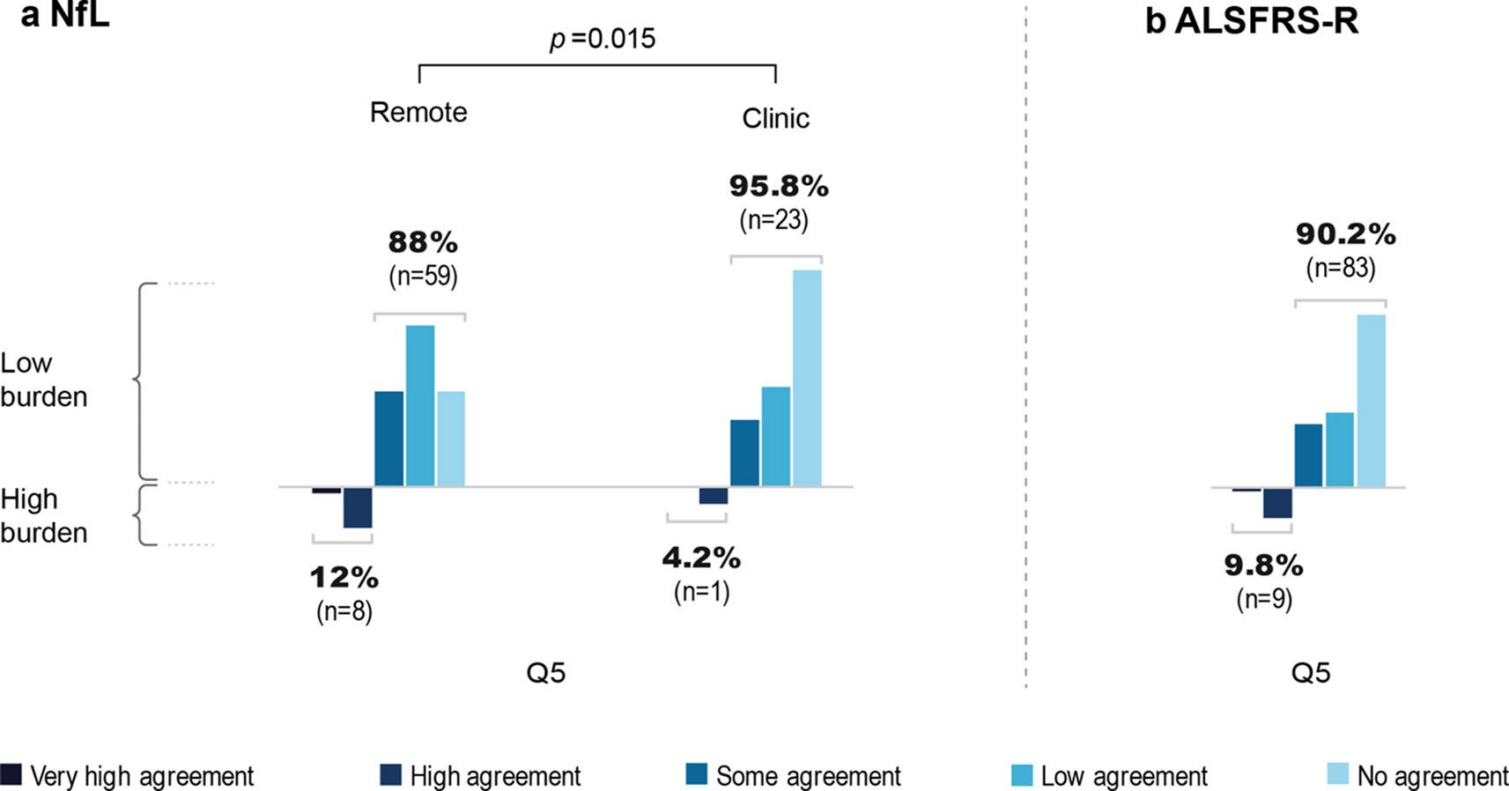
Burden due to NfL and ALSFRS-R. **a** NfL. Patients with a known NfL value (*n* = 91) were asked about the burden caused by NfL communication: **Q5** “Do you perceive burden caused by the NfL?”. A distinction was made between a “remote information group” with NfL remote notification (*n* = 67) and a “clinical information group” (*n* = 24) with NfL clinic notification. **b** ALSFRS-R. Patients with previous ALSFRS-R remote digital assessment (*n* = 92) were asked about the burden caused by this procedure: **Q5** “Do you perceive the self-assessment of the ALSFRS-R as a burden?”. *n* Number of patients; *NfL* Neurofilament Light Chain; *ALSFRS-R* ALS Functional Rating Scale Revised

Information about NfL was shared with 91 patients. Burden by information about NfL was perceived in 4.2% (*n* = 1) and 12% (*n* = 8) of patients in the clinic and remote information groups, respectively. The difference between the clinic and remote information groups was statistically significant (*p* = 0.015). Remote digital assessment (*n* = 92) of the ALSFRS-R was perceived as burdensome in 9.8% of cases (*n* = 9). Results are shown in Fig. 4.

### Fear of progression (FoP)

**Fig. 5:**
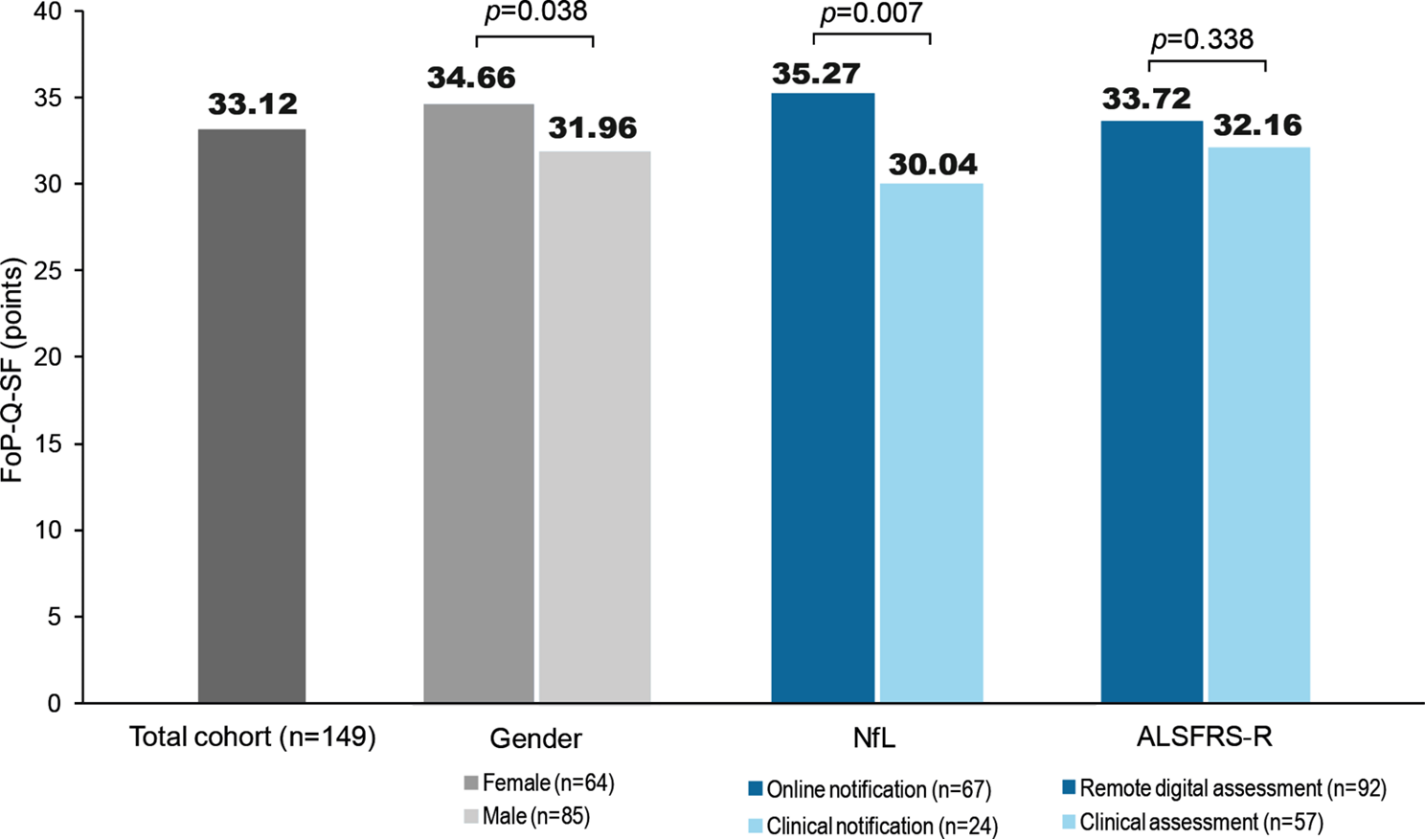
Fear of Progression (FoP). Based on 12 questions on various concerns about the future, a total score of the Fear of Progression Questionnaire – Short Form (FoP-Q-SF) was formed, with a possible value between 12 (no anxiety) and 60 points (very severe anxiety). The mean FoP values are presented in the diagram. Fear of disease progression (FoP) was defined from a cut-off value of ≥ 34 points. *n* Number of patients; *NfL* Neurofilament Light Chain; *ALSFRS-R* ALS Functional Rating Scale Revised

The mean FoP-Q-SF score was 33.12 (± 8.88) out of a possible 60 points. A total of 40.3% of respondents (*n* = 60) met the criteria for severe fear of progression (FoP) with a score of ≥ 34 points. Women had higher FoP values than men and the difference was statistically significant (*p* = 0.038). Patients with remote information about NfL levels had significantly higher FoP scores than patients who were informed in the clinic (*p* = 0.007). In the remote NfL group (*n* = 67), 56.7% of patients (*n* = 38) exhibited severe FoP, compared to 20.8% (*n* = 5) and therefore less in the clinical NfL group (*n* = 24). There was no difference between patients with remote and clinic assessment of the ALSFRS-R (*p* = 0.338) (Fig. 5).

## Discussion

This study focused on patients’ perception of shared information about NfL and ALSFRS-R, which are among the main prognostic factors of disease progression in ALS [27, 28]. The results of this study show that NfL and ALSFRS-R were experienced as equally important for ALS research (98 and 96%, respectively). This observation came as no surprise, as both parameters were developed primarily in a research context. The finding that almost all patients recognized a value for research in these prognostic factors is encouraging for the status of these outcome parameters, as researchers, regulators and patients themselves clearly agree on the utility of these endpoints. The proportion of participants who were able to derive a personal benefit from shared information about NfL (75%) and ALSFRS-R (77%) was remarkably high, underscoring its evolution from research-only parameters to clinical practice data being embraced by patients. Only 24 of 57 NfL tested patients received an information about the NfL result in the clinical setting, indicating that sharing this information is not yet standard practice. Nevertheless, 83.2% of all patients expressed a strong interest in NfL, and 75.2% considered this information personally relevant. These findings suggest that sharing such prognostic data could be highly beneficial for patients.

The importance of this study lies in the comprehensive investigation of patient perspectives on two important prognostic factors in ALS, NfL and ALSFRS-R. Both prognostic parameters are used as primary and secondary endpoints [29, 30] as well as stratification criteria in clinical trials. Given the strong correlation between NfL elevation and the ALS progression rate, NfL is increasingly considered as prognostic criterion in clinical practice [3, 31]. Also, the results of ALSFRS-R assessments are applied in the decision-making in ALS care [32].

The NPS results of this study revealed a strong recommendation of NfL (+ 21) and ALSFRS-R (+ 26) to fellow patients and contributed to the notion of an overall positive perception of information sharing of the two investigated prognostic factors. The positive perception from the patient perspective supports the ongoing concept of sharing NfL and ALSFRS-R information between health care professionals and patients alike, and, moreover, making it available remotely.

Before the study, there was uncertainty about a potential burden of sharing prognostic data. Various knowledge sources, including internet publications and podcasts, allow ALS patients to independently access information about NfL and ALSFRS-R [33]. Alternatively, patients receive personalized information during visits to specialized ALS outpatient clinics. During these consultations, the benefits of biomarker assessment and potential misconceptions are carefully addressed [34]. In this cohort, only a small group experienced a burden in the clinical sharing of the NfL biomarker (4.2%), with a significantly greater burden when shared remotely (12%). Remote digital assessment of the ALSFRS-R was well received, with a reported burden in 9.8% of the participants. However, the absolute frequencies of patients who experienced distress when sharing remote access were very low. To prevent patients from unwanted prognostic information, patient’s preferences must be integrated as part of information management. Furthermore, the integration of patient-centric supplementary information such as frequently-ask-questions (FAQ) via the ALS app, can contribute to reduce the burden due to prognostic information and to prevent miscomprehension of test results. Furthermore, personalized data presentation on the app will contribute to further reducing or preventing burdensome medical information. Accordingly, patients will have the option of deactivating the NfL diagram in the app in the future.

This study identified a high level of fear of progression (FoP) among the patients surveyed, revealing their self-reflection on the progressive character of the disease. With a prevalence of 40%, FoP level was significantly higher than in cancer patient cohorts, where it was defined using the same cut-off value [35]. Women typically exhibit higher FoP levels than men, a finding that was also observed in our study (*p* = 0.038) [24]. However, given the larger proportion of men in our study cohort, the results cannot be explained by gender distribution. A comparison of the clinic and remote subgroups showed greater FoP in the remote information group, whose individual NfL values were displayed on a mobile application named “ALS app” (*p* = 0.007). Conversely, FoP scores were not increased in patients with remote digital assessment of the ALSFRS-R (*p* = 0.299). Therefore, conclusions about a correlation between remotely shared information and FoP are premature and need to be further investigated. However, the existing data showed that despite the high level of FoP, the perceived benefit for sharing information about NfL and ALSFRS-R was high. Patients could benefit from a low-barrier access to such prognostic information, as it enables them to make informed decisions throughout the course of ALS, although the remote transmission of such data may increase FoP.

Despite the wide use of NfL and ALSFRS-R by neurologists and other health care professionals, the patients’ perspective on these prognostic parameters has not yet been studied. The inclusion of the patient’s view is relevant, as patients gain increasing access to this prognostic information [14]. A strength of this study is the combined investigation of a biomarker (NfL) and a patient-reported outcome (ALSFRS-R), and the comparison of patients in a conventional clinical setting with participants in the (additional) remote setting of information sharing using the ALS app. However, the study results should be viewed in the context of their limitations. The sample size was relatively small, and the groups with remote vs. clinic information were not equally sized or distributed. Thus, differences between groups (NfL vs. ALSFRS-R, clinical vs. remote setting) may be underestimated and need to be studied in larger cohorts. It should be noted that SOD1-associated ALS patients receiving Tofersen treatment were included in this study. These patients may have rated NfL more positively, as Tofersen is associated with a strong reduction of NfL [30, 36]. Caution is warranted when transferring the NPS system – being optimized for validating consumer products and services – to medical information [22, 37]. A further limitation of the NPS should be noted as the NPS values for NfL and the ALSFRS-R currently stand alone and cannot yet be compared with other prognostic factors or other cohorts.

## Conclusions

In conclusion, this study underscored that patients value information about NfL and ALSFRS-R, not only for their own understanding of the disease, but also for its relevance for ALS research. The perceived burden by prognostic information was found to be low in most patients, so that sharing data on NfL and ALSFRS-R – in personal contact or remotely – is justified. Future studies with larger cohorts, longer observation intervals and the inclusion of additional prognostic factors, such as the ALS progression rate and vital capacity, will contribute to further improve the concept of patient-centered information sharing in ALS.

## Data Availability

The datasets used and analyzed during the current study are available from the corresponding author on reasonable request.

## Abbreviations

ALS: Amyotrophic Lateral Sclerosis
NfL: Neurofilament Light Chain
ALSFRS-R: Amyotrophic Lateral Sclerosis Functional Rating Scale Revised
FoP: Fear of Progression
FoP-Q-SF: Fear of Progression Questionnaire – Short Form
